# Psychosocial Correlates of Fatigue in Young Adults with Multiple Sclerosis: Exploring the Roles of Resilience, Mindfulness, and Illness Perception

**DOI:** 10.3390/healthcare13182335

**Published:** 2025-09-17

**Authors:** Silvia Poli, Valeria Donisi, Roshan das Nair, Maria Angela Mazzi, Alberto Gajofatto, Michela Rimondini

**Affiliations:** 1 Section of Clinical Psychology, Department of Neuroscience, Biomedicine and Movement Science, University of Verona, 37134 Verona, Italy; silvia.poli@univr.it (S.P.); mariangela.mazzi@univr.it (M.A.M.); michela.rimondini@univr.it (M.R.); 2 Department of Health Research, SINTEF Digital, 7030 Trondheim, Norway; roshan.nair@sintef.no; 3 School of Medicine, University of Nottingham, Nottingham NG8 1BB, UK; 4 Section of Neurology, Department of Neuroscience, Biomedicine and Movement Science, University of Verona, 37134 Verona, Italy; alberto.gajofatto@univr.it

**Keywords:** multiple sclerosis, fatigue, mindfulness, young adults, illness perception, resilience, anxiety, depression, health psychology

## Abstract

**Background and Objectives**: Fatigue, despite being one of the most common and disabling symptoms in multiple sclerosis (MS), is far from being fully understood. The aim of the present study was to explore the association between fatigue and resilience, illness perception, and mindfulness traits, accounting for the impact of anxiety and depression in young adults with MS (YawMS). **Methods**: For this cross-sectional exploratory analysis, the following inclusion criteria applied: age 18–45 years, MS diagnosis, Expanded Disability Status Scale <3.5. Fifty-one YAwMS (mean age: 33.5 ± 6.7 years; 76% women, 24% men; 96% relapsing-remitting MS) completed validated questionnaires. Student’s t-tests and Spearman correlations, with partial correlations controlling for anxiety and depression, were performed. Finally, a preliminary multivariate model (seemingly unrelated regression) was applied. **Results**: Despite low disability levels, 69% experienced moderate to severe fatigue (average fatigue score 61.9 ± 17.9). Higher total fatigue was associated with negative illness perception, particularly regarding identity and consequences (*p* = 0.66 and *p* = 0.67, respectively), and lower levels of non-judgment and non-reactivity (*p* = −0.48 and *p* = −0.54, respectively), and these relationships persisted after controlling for anxiety and depression. Although resilience was negatively correlated with fatigue, its impact was not maintained. **Conclusions**: Our findings emphasize the relevance of fatigue in YawMS with low disability levels. Cognitive and emotional processing might be associated with fatigue, beyond and beside disease severity itself.

## 1. Introduction

Fatigue can be described as a lack of energy, an overwhelming sense of tiredness, or a feeling of exhaustion that is commonly experienced by people living with different chronic illnesses [1], such as neurological diseases [2], cancer [3], cardiovascular disease [4], fibromyalgia [5], chronic kidney disease [6], and asthma [7]. Contrary to what happens in healthy people, fatigue in chronically ill patients is experienced independently from physical activity, is not relieved by rest, and has a significant impact on daily life [8].

Fatigue is one of the most common and disabling symptoms reported by people with multiple sclerosis (MS) [9,10,11], a chronic disease usually first diagnosed at the age of 20–40 years [12]. Although a consensus on a definition of fatigue has not been reached in the context of MS [13], clinical guidelines refer to it as “a subjective lack of physical and/or mental energy that is perceived by the individual or caregiver to interfere with usual and desired activities” [14].

MS-related fatigue is a symptom that is far from being fully understood, and its causes are still unknown [8,15]; however, primary and secondary mechanisms have been described. Primary mechanisms are directly related to the physiopathology of MS (e.g., neuronal circuits, and immunological pathways) [13,16], while secondary causes refer to non-disease-specific and psychosocial factors such as impaired sleep, depressive symptoms, cognitive impairment, and physical deconditioning [16,17]. The complex interplay of these factors can be conceptualized through the biopsychosocial model, which emphasizes the mutual interactions among biological, psychological, and social aspects of an illness [18,19]. This model has been applied to investigate fatigue in different chronic diseases (e.g., rheumatoid arthritis [20]), including MS [21,22].

According to a recent literature review, the prevalence of fatigue in adults with MS ranges from 36.5 to 78% [23], and a large national study in Norway found that 81% of people with MS had at least mild fatigue [24].

In addition to its high prevalence, fatigue has been reported to be the most bothersome symptom in MS [25] and has a significant impact on patients’ functioning [26]. MS-related fatigue is one of the main determinants of quality of life [27], with fatigued patients consistently reporting lower quality of life [23]; fatigue has also been found to positively correlate with anxiety and stress [24,28].

Social participation [29] and physical activity levels [8] are also negatively impacted by fatigue. In qualitative research, people with MS have described feelings of guilt and frustration about the limitations imposed on their lives by fatigue. Moreover, they have reported fear of being perceived as lazy if fatigue prevented them from meeting work and social commitments [30]. In fact, fatigue is often referred to as an ‘invisible symptom’ [31] because, despite being one of the most debilitating aspects of the disease, it remains challenging for individuals who do not directly experience it to perceive and observe it. This lack of visibility makes it difficult for patients to feel understood, leading to frustration and isolation, while also complicating their ability to seek and receive appropriate support. Fatigue has also been reported to be a reason for treatment discontinuation [32] and to cause dependency and loss of valued roles, including work [30,33]. The economic impact of fatigue is, in fact, also relevant. There is a significant association between the presence or severity of fatigue and employment outcomes such as employment status, capacity to work, and sick leave [8,23]. Absenteeism and presenteeism, which have been linked to anxiety, relapses, and fatigue, are common phenomenon even in earlier stages of the disease and in cases of low physical disability [34,35].

Young adults with MS (YawMS) are affected by fatigue, as this symptom is reported since the early stages of MS [36,37] or even years before being diagnosed with MS [38]. Moreover, lower age is one factor that has been associated with fatigue interference [39]. From a psychological perspective, this is because managing fatigue can be particularly challenging for younger individuals, who often manage significant family and work responsibilities. Additionally, since fatigue is not commonly associated with younger people, individuals may struggle to disclose and accept it. However, YawMS are a subgroup that has received little attention in MS research [40]. According to the few studies on this population, young adults with MS have high levels of depression and negative affect [41] and low levels of resilience [42].

The literature suggests the importance of identifying non-pharmacological lifestyle factors to advance treatment strategies [15]. Gaining more knowledge of the psychosocial factors related to fatigue in this specific population would help to design better interventions to alleviate and manage fatigue in the early stages of MS. This would improve well-being, help YawMS to maintain employment, and enable them to live more sustainable lives.

The aim of the present study was to (i) describe fatigue in a sample of young adults with MS and (ii) investigate the association between fatigue and psychosocial factors (resilience, illness perception, and mindfulness traits). Considering the above-mentioned literature that highlights the association between fatigue and anxiety and depression, we considered them as confounders and (iii) preliminarily explore the combined influence of these psychosocial factors on both motor and cognitive dimensions of fatigue.

## 2. Materials and Methods

### 2.1. Participants

Participants were recruited at the Multiple Sclerosis Clinic Center of Verona University Hospital (Regional Referral Multiple Sclerosis Center, Verona, Italy) and provincial MS center clinics [43,44]. The study is part of a larger project named ESPRIMO that started in 2018 at the Department of Neuroscience, Biomedicine, and Movement of the University of Verona in collaboration with the Verona University Hospital Trust. The project has been approved by the Ethical Committee for Clinical Trials of the Provinces of Verona and Rovigo (Prog 2676CESC) and was performed according to the latest version of the Declaration of Helsinki. Informed consent was obtained from all participants involved in the study.

In the current paper, secondary analyses from the baseline dataset of the ESPRIMO intervention feasibility study are presented. In the study persons with MS were enrolled according to the following inclusion criteria: young adults (age range 18–45 years), MS diagnosis as reported by the treating neurologist in medical records according to the revised McDonald Criteria [45], Italian speakers, and willing and able to offer signed informed consent. Exclusion criteria were clinically relevant cognitive deficits (assessed by the treating neurologist) that could have been an obstacle in completing the questionnaires, major psychiatric disorders (e.g., psychosis, bipolar disorder, substance abuse disorders, dissociative disorders, or a current diagnosis of major depression) as assessed by the treating neurologist or the clinical psychologist, and an Expanded Disability Status Scale (EDSS) score above 3.5 [46].

Using a purposive sampling approach, eligible participants were contacted by clinical psychologists working at the Clinical Psychology Unit of the Verona University Hospital after being informed by neurologists or residents working at the MS centers.

Out of 69 patients who consented to participate, fifty-one persons with MS completed the baseline assessment of the intervention evaluation and thus were included in the current paper.

### 2.2. Measures

Sociodemographic characteristics (e.g., gender, educational level, occupation, and living situation) were collected using a bespoke questionnaire; information on the type of MS, disease duration, pharmacological treatment, and EDSS was compiled by the neurologist from the patients’ clinical case sheets. The following variables were collected using validated measures: fatigue, mindfulness traits, illness perception, anxiety, and depression.

To assess fatigue, we used the Fatigue Scale for Motor and Cognitive Functions (FSMC) [47], a self-report fatigue questionnaire validated in MS patients. It consists of 20 items assessed on a Likert scale ranging from 1 (never happens) to 5 (always happens), with higher scores reflecting higher levels of fatigue. FCMC provides a score for cognitive, motor, and total fatigue. Cut-off scores [47] are as follows: cognitive: 10–21 absent, 22–27 mild, 28–33 moderate, 34–50 severe fatigue; motor: 10–21 absent, 22–26 mild, 27–31 moderate, 32–50 severe fatigue; total: 10–42 absent, 43–52 mild, 53–62 moderate, 63–100 severe fatigue.

The Connor Davidson Resilience Scale (CD-RISC 25) [48,49,50,51] consists of 25 items, each rated on a 5-point scale (from 0 ‘not at all true’ to 4 ‘almost always true’). The total score ranges from 0 to 100, with higher scores indicating higher resilience. CD-RISC 25 has been used in MS research [51] and also in Italy [52].

To describe mindfulness traits, the Italian version of the Five-Facet Mindfulness Questionnaire (FFMQ) [53] was used. The FFMQ is a 24-item questionnaire that measures five aspects on a 5-point Likert scale, from 1 (“never or very rarely true”) to 5 (“very often or always true”). Higher total scores reflect a higher degree of mindfulness. The FFMQ measures five dimensions, defined as facets: observe (notice and observe internal/external experiences), describe (express thoughts and emotions in words), act with awareness (engage in actions proactively and not instinctively), non-judge (observe internal states suspending judgment), and non-react (refrain from immediate reactions to emotions). A total score was calculated by adding the score from each facet. It has good psychometric properties that support its use in the Italian context [54] and has been used with MS patients [55].

Illness perception was assessed using the Brief Illness Perception Questionnaire (B-IPQ) [56,57], a 9-item instrument developed to provide a quantitative measurement of the emotional and cognitive representations of illness. One question is open-ended and investigates causes. The other eight dimensions are evaluated on a 5-point Likert scale (ranging from “strongly disagree” to “strongly agree”). They assess the symptoms experienced (identity), the illness impact on patients’ lives (consequences), the perception of the length of the disease (timeline), the ability to control MS through one’s actions (personal control), the effectiveness of therapies in controlling MS (treatment control), the level of concern about the disease (concern), the extent to which MS is comprehensible to the patient (coherence), and the negative emotions associated with the illness (emotional response). Higher scores reflect a more negatively perceived illness.

Anxiety and depression symptoms were assessed with the Italian validation [58] of the Hospital Anxiety and Depression Scale (HADS) [59]. HADS is a brief self-report questionnaire consisting of 14 items describing on a 4-point scale from 0 to 3 the level of anxiety (HADS_A; 7 items) and the level of depression (HADS_D; 7 items) the person is experiencing. Cut-off scores are: 0–7 for no symptoms, 8–10 for mild symptoms, 11–14 for moderate symptoms, and 15–21 for severe symptoms.

### 2.3. Statistical Analysis

Descriptive statistics are presented as mean values and standard deviation (SD) for continuous variables and as frequencies or percentages for categorical variables. One-sample t-tests were used to explore the differences between our sample and the sample of the Italian validation study (where available). Correlational analyses between fatigue and psychological variables (illness perception, mindfulness, and resilience) were performed using Spearman’s rank correlation, relaxing the linearity assumption. Anxiety and depression are known to be associated with fatigue; therefore, we considered them as confounders using partial correlation.

Finally, a set of models, based on the seemingly unrelated regression approach assuming correlated errors, was applied to explore the joint effect of multiple explanatory variables on the outcome, measured with two correlated scales: motor and cognitive fatigue. The parsimony criterion was used to select the explanatory variables after checking for multicollinearity among them using the variance inflation factor (VIF), calculated by Stata’s collin command. Values below 10 are considered as indices of low collinearity. The selection was performed in two steps: first, the potential confounding variables were checked, then psychosocial scores were individually tested and, if statistically relevant, added to the final model.

Since the sample size is small and the models likely violate the assumption of normality of the error distribution, residual bootstrap estimators were applied with 500 replications [60]. This robust technique optimizes the standard errors of the regression parameters. The standard bootstrap approach, indeed, resamples the original dataset before calculating the regression, while residual bootstrap first estimates the regression and then randomly replicates its residuals.

All the analyses were performed with STATA 18 [61].

## 3. Results

### 3.1. Sociodemographic, Clinical, and Psychological Characteristics

A total of 51 people with MS were included in the analyses. As reported in Table 1, most of the participants were women (76.5%), employed (68.6%), and had a graduate or post-graduate degree (49%). The mean age was 33.5 ± 6.7 years (range 22 to 45 years). Almost all (96%) had a diagnosis of relapsing-remitting multiple sclerosis (RRMS), with two persons having a diagnosis of primary progressive MS (PPMS). Average time since diagnosis was 6 years (SD = 5.8); however, 23.5% received the diagnosis within 1 year of the study. Most (69%) had an EDSS score between 0 and 1.5 (see Table 1).

Mean scores of the psychological variables are presented in Table 2.

Regarding anxiety and depression symptoms (HADS), no differences were highlighted between our sample and the Italian validation sample (oncological patients) [58]. According to the cut-off score, 90% presented no or mild levels of depressive symptoms, while 10% presented moderate levels. Regarding anxiety, 78% presented no or mild anxiety, 18% presented moderate levels, and 4% presented severe levels. The Spearman correlation between anxiety and depression is 0.51.

The B-IPQ_TOT in our sample was significantly higher than that of an Italian sample (age: 44.6; EDSS: 2.3) of people with MS (*t* = −2.86; *p* < 0.01) [62].

Resilience (CD-RISC_TOT) had a mean score of 60.12 ± 15.7, which is lower (*t* = −10.48; *p* = 0.00) than that of the validation study sampling the US general population [50].

Regarding the Five Facet Mindfulness Questionnaire (FFMQ), the subscales observe (*t* = −4.06; *p* < 0.001), describe (*t* = −2.46; *p* < 0.05), and awareness (*t* = −3.70; *p* < 0.001) were significantly lower than the sample of Italian adults (general population) [63]. The other two subscales were not significantly different.

### 3.2. Fatigue

The mean score of the total fatigue scale was 61.9 ± 17.9 (range 23–97; *sk* = −0.11, *k* = 2.3, *sk* test = 1.87, *p* = 0.39). Cognitive fatigue had a mean of 29.7 ± 9.4 (range 10–49; *sk* = 0.1, *k* = 2.3, *sk* test = 1.90, *p* = 0.39), while motor fatigue had a mean of 32.2 ± 9.5 (range 13–48; *sk* = −0.2, *k* = 2.1, *sk* test = 4.68, *p* = 0.10). In particular, 57.7% of YawMS had moderate to severe cognitive fatigue, and 68.7% had moderate to severe motor fatigue (see Table 3).

### 3.3. The Association Between Fatigue and Sociodemographic Characteristics and Psychological Variables

No statistically significant differences were found for age and time since diagnosis for cognitive, motor, and total fatigue, but men had lower total, motor, and cognitive fatigue compared to women (see Table 4; normality assumptions are presented in Appendix A.1).

Table 5 shows correlational analysis between fatigue subscales (i.e., motor fatigue and cognitive fatigue) and B-IPQ (illness perception), FFMQ (mindfulness) and CD-RISC 25 (resilience) items; partial correlation with HADS_A and HADS_D are also presented.

**Figure 1:**
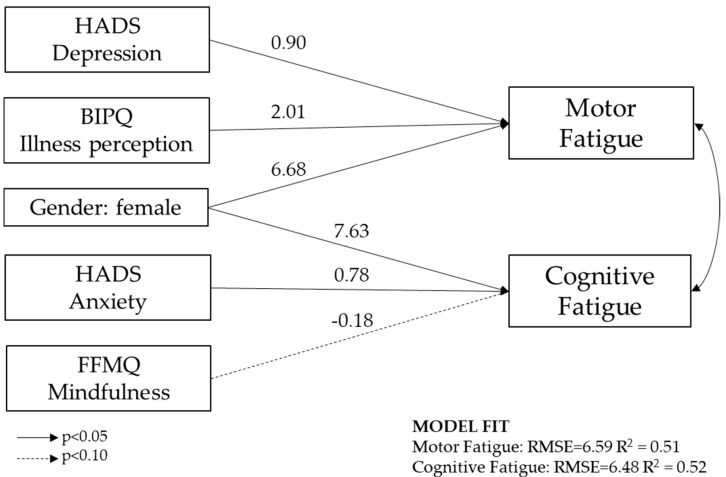
Path analysis model based on seemingly unrelated regression predicting motor and cognitive fatigue, as measured by the Fatigue Scale for Motor and Cognitive Functions. Rectangles represent observed variables assessed through standardized questionnaires (HADS = Hospital Anxiety and Depression Scale; BIPQ = Brief Illness Perception Questionnaire; FFMQ = Five Facet Mindfulness Questionnaire). Arrows represent standardized path coefficients; model fit, namely the coefficient of determination (^R^^2^) and Root Mean Square Error (RMSE), is shown in the figure.

Finally, we jointly examined the impact of psychosocial variables and gender on the outcomes, namely motor and cognitive fatigue. Figure 1 presents the final model. Female gender, depressive symptoms, and illness perception were significantly associated with motor fatigue, while female gender and anxiety were significant predictors of cognitive fatigue. Mindfulness showed a negative association with cognitive fatigue, though less relevant.

The model explained approximately 52% of the variance in both motor and cognitive fatigue (*R*^2^ = 0.51 and *R*^2^ = 0.52, respectively). The residual correlation is 0.58, showing a dependency between outcomes (Breusch-Pagan test of independence *chi*^2^(1) = 17.3, *p* < 0.01). Details of the regression coefficients and bootstrap estimates are reported in Table 6, while the collinearity diagnostics are presented in Appendix A.2 (specifically, VIF ranges from 1.16 to 1.84, and the mean VIF is 1.53).

## 4. Discussion

The present study aimed to describe fatigue in a sample of young adults living with MS and to explore its association with psychosocial factors (i.e., resilience, illness perception, and mindfulness traits) involved in the SM process of adaptation, also considering anxiety and depression symptoms.

Despite the relatively low presence of anxiety and depression symptoms, the young age, and the low disability, participants reported significant levels of fatigue.

Half of our participants reported having severe fatigue, with severe motor fatigue being more prevalent (57%) than severe cognitive fatigue (36%).

Comparing our results with studies using the same scale of fatigue, our sample had higher levels of fatigue for total fatigue and both cognitive and motor fatigue compared to a Finnish sample of people with MS with an average older age (50.9) and a moderate disability (as measured by a self-reported EDSS of 4.8) [64]. Our sample’s fatigue scores were also higher than in an Austrian sample with a comparable mean age (39.5) and low disability (mean EDSS 1.5) [65]. The higher fatigue levels in our sample compared to other studies suggest that fatigue is not solely linked to disease-related variables (e.g., disability levels) but might also be influenced by individual factors (e.g., coping mechanisms) or sociocultural aspects. While few studies have specifically addressed the socio-cultural dimensions of MS-related fatigue, research in the general population indicates that fatigue can be impacted by different variables such as socio-economic status [66] and cultural norms, which might influence how individuals perceive and report fatigue [67]. Moreover, the organization of healthcare systems, including policies, resources, and service accessibility, can influence access to support (e.g., including fatigue management intervention). Future studies should investigate these relationships more directly.

Regarding the association between fatigue and sociodemographic and clinical variables, previous studies have found mixed results on EDSS, with some studies finding a correlation between EDSS and fatigue [68,69], while others reporting that fatigue is not necessarily associated with the level of disability or disease variant (EDSS-based benign vs. non-benign MS) [70]. The latter findings are in line with the results of our study, highlighting that the EDSS might not fully capture the complexity of disability in PwMS. In fact, the scale primarily focuses on walking ability while overlooking other relevant factors such as cognitive function, mood, and overall quality of life, which may play a crucial role in fatigue perception. In the literature, conflicting evidence has also been found for age [71,72], which was not a significant variable for fatigue in our sample.

Such discrepancies underscore the challenges both in assessing fatigue and in comparing results across studies. A variety of measurements and cut-off values are available for assessing fatigue, yielding variable estimates of fatigue prevalence. Moreover, available self-report questionnaires often lack construct validity and measure different but overlapping constructs, such as depression or pain [13,73].

Our findings revealed interesting associations between fatigue and psychosocial factors that were tested, accounting for anxiety and depression.

Specifically, illness perception was associated with fatigue, which aligns with a recent study’s findings [74]. We found that perceived consequences and illness identity correlated with cognitive and motor fatigue. This suggests that individuals who perceive the impact of MS to be severe and who strongly experience symptoms may be more vulnerable to experiencing heightened fatigue levels, with the association being stronger for motor fatigue. Importantly, these associations remained significant even after controlling for anxiety and depression, indicating that the way patients perceive their illness has an independent association with their fatigue experience.

The preliminary multivariate model showed a contribution of illness perception on motor fatigue, along with depression and anxiety. Interestingly, the cognitive and emotional representation of the illness did not contribute significantly to cognitive fatigue. These findings suggest that different dimensions of fatigue may be associated with distinct psychological mechanisms, highlighting the need for further research into the specific roles of psychological factors within the context of fatigue management.

Examining facets of mindfulness, correlational results point to the particular relevance of the non-judgment (taking a non-evaluative stance toward internal thoughts and feelings) and non-reactivity (the ability to allow emotions and thoughts to flow without acting or interfering) dimensions, which had an association with fatigue independent from anxiety and depression.

Interestingly, the ability to observe internal and external experiences was not associated with fatigue. This finding is in contrast with some studies where the “Observe” facet mediated the effects of mindfulness-based interventions in patients with diabetes [75] and psychosis [76]. Further research is needed to explore the mechanism underlying improvement in fatigue in Cognitive Behavioural Therapy and mindfulness-based treatments and to determine whether emphasizing specific mindfulness facets could enhance intervention effectiveness for fatigue management in MS.

The ability to label internal experiences (describe) and to attend to what is happening in the present (awareness), despite being significantly lower in our sample compared to that of the general Italian population [63], showed relatively weak associations with fatigue. Specifically, the correlations of “Describe” and “Awareness” with fatigue were minimal and became negligible after controlling for anxiety and depression. This suggests that while describe and awareness are important aspects of mindfulness, they may not directly mitigate fatigue symptoms in young adults with MS. Instead, these facets might be associated with fatigue indirectly or in conjunction with other psychological factors. A recent study concluded that the relationship between trait mindfulness and fatigue is mediated by depression [77]. In our model, mindfulness showed a negative association with cognitive, but not motor, fatigue. Future studies on larger samples could investigate more complex models, including possible mediation pathways between negative effect, specific mindfulness facets, and cognitive and motor fatigue.

Similar to some other mindfulness facets, resilience, whose effect on fatigue diminished when anxiety and depression were controlled for, might have a buffering effect mediated by overall emotional well-being. In line with this hypothesis, a study found positive affect to be a mediator between fatigue intensity and resilience, while fatigue catastrophizing was not directly related to resilience [78]. High resilience scores are associated with fewer symptoms of depression and anxiety [79]. Resilience could, therefore, indirectly affect fatigue, and its protective effect alone may not be sufficient to counteract the negative impact of emotional distress.

Managing fatigue is described in the literature as a constant challenge by people with MS, and clinicians should prioritize the promotion of the ability to manage fatigue in people with MS [30]. Our results highlight the relevance of fatigue management for young patients.

Pharmacological treatments for MS fatigue have demonstrated little to no efficacy, leading to advice against using them indiscriminately [13,73]. Non-pharmacological interventions such as psychological interventions or physical activity are currently considered the preferred treatment and have demonstrated good effects on fatigue [13,16,80,81]. In particular, recent reviews and studies have suggested the effectiveness of mindfulness-based interventions in reducing fatigue in people with MS [82,83,84]. Our findings support the notion that mindfulness is associated with fatigue and preliminarily suggest a possible mechanism of action for mindfulness-based interventions via improved acceptance and non-judgmental attitude. This hypothesis should be verified in a larger sample and using a longitudinal design. Nevertheless, these observations may provide a basis for future studies to investigate Acceptance and Commitment Therapy, a third-wave therapy specifically focused on acceptance, whose effect on fatigue management has received preliminary positive, though limited, evidence [85].

### Limitations

Some limitations of the study should be taken into consideration. First, its cross-sectional design precludes any inference of causality between fatigue and the psychosocial factors examined. Second, the representativeness of our findings might be impacted by the relatively small sample and selection bias; participants, in fact, volunteered to participate in an intervention and, therefore, could represent a more health-conscious or in-need subgroup that may not fully reflect the broader MS population. Additionally, sampling bias could also be incurred. Because our focus was on young adults with low disability, which constitutes an understudied group, it limits the external validity and generalizability of our findings to broader MS populations, particularly older patients or those with more advanced disease stages. Third, the lack of a control group (e.g., with healthy individuals) precludes comparative interpretations. Lastly, despite the use of a parsimonious modeling strategy and robust estimation methods, the multivariate model should be considered preliminary due to the modest sample size. However, the model offers insights into the relationship between psychological variables and fatigue and warrants replication in larger samples to validate the observed associations and to test the interactions between variables in determining fatigue levels.

## 5. Conclusions

Young adults with MS had negative illness perception and high levels of fatigue despite having a low disability level. Therefore, our findings emphasize the relevance of fatigue in young adults with MS. Fatigue is a complex symptom that might be intricately related to patients’ cognitive and emotional processing of the disease, beyond and beside disease severity itself. This study adds to the growing literature on fatigue, testing the associations with psychological variables other than anxiety and depression, and preliminarily suggesting their combined and differential effect on cognitive and motor fatigue. Future studies with larger and more diverse samples are needed to replicate and extend these findings. Although further replication is needed, the current results highlight the potential value of treatment approaches that focus on reshaping maladaptive illness perceptions and promoting mindfulness, which could prove particularly effective in managing fatigue. In particular, future studies should specifically focus on helping people improve their levels of acceptance and non-judgmental attitude as a way of improving fatigue perception, with the ultimate goal of enhancing quality of life and facilitating better social and occupational functioning.

## Figures and Tables

**Table 1:** Sociodemographic and clinical characteristics of the participants (*n* = 51).

Variable	*n* (%)
*Gender*	
Female	39 (76)
Male	12 (24)
*Educational status*	
Secondary school/professional school degree	11 (21.6)
High school degree	14 (27.5)
Graduate or post-graduate degree	25 (49)
Missing	1 (1.9)
*Occupation*	
Employed	35 (68.6)
Unemployed	6 (11.8)
Student	3 (5.9)
Housewife	3 (5.9)
Retired	1 (2)
Other	3 (5.9)
Age: mean ± SD	33.5 ± 6.7
Time since diagnosis: mean ± SD	6.2 ± 5.8
*Disease duration*	
Up to 1 year	12 (23.5)
2–3	7 (13.7)
4–5	7 (13.7)
6–11	24 (47.1.)
≥12	8 (15.7)
Missing	1 (1.9)
*Multiple sclerosis type*	
Relapsing remitting	49 (96.1)
Primary progressive	2 (3.9)
*EDSS*	
0	11 (21.6)
1	12 (23.5)
1.5	12 (23.5)
2	4 (7.8)
2.5	3 (5.9)
3	3 (5.9)
3.5	6 (11.8)

**Table 2:** Mean scores of anxiety, depression, illness perception, resilience, and mindfulnesss facets.

Variable	Mean ± SD	Skewness	Kurtosis	Test for Normality (*p*-Value)
**Anxiety**				
HADS_A	8.2 ± 3.9	0.7	3.4	5.45 (0.07)
**Depression**				
HADS_D	5.0 ± 3.4	0.6	2.4	4.5 (0.11)
**Illness perception (B-IPQ)**				
Consequences (B-IPQ 1)	5.3 ± 2.4	−0.1	1.8	8.9 (0.01)
Timeline (B-IPQ 2)	9.3 ± 1.6	−3.3	15.6	43.1 (<0.01)
Personal control (B-IPQ 3)	5.2 ± 2.1	0.1	2.8	0.22 (0.90)
Treatment control (B-IPQ 4)	7.9 ± 2	−0.9	3.4	7.51 (0.02)
Identity (B-IPQ 5)	4.9 ± 2.4	<0.1	1.9	6.64 (0.04)
Concern (B-IPQ 6)	6.5 ± 2.3	−0.2	2.3	2.18 (0.34)
Coherence (B-IPQ 7)	6.7 ± 2.1	−0.3	2.4	1.59 (0.45)
Emotional response (B-IPQ 8)	6.4 ± 2.8	−0.6	2.2	5.16 (0.08)
Total score (B-IPQ TOT)	45.8 ± 10.1	−0.4	2.8	2.02 (0.36)
**Resilience (CD-RISC)**	60.12 ± 15.7	−0.2	2.1	4.34 (0.11)
**Mindfulness (FFMQ**)				
Observe	13.7 ± 4	−0.3	2.4	2.13 (0.35)
Describe	16.2 ± 3.5	−0.2	2.8	0.59 (0.75)
Awareness	16.9 ± 3.9	<0.1	3.0	0.22 (0.90)
Non-judge	15.6 ± 4.2	−0.2	2.4	1.38 (0.51)
Non-react	13.6 ± 3.7	<0.1	2.6	0.18 (0.91)
Total score FFMQ	75.9 ± 11.3	0.1	3.1	0.41 (0.81)

**Table 3:** Distribution of level of cognitive fatigue, motor fatigue, and total fatigue.

**FSMC cognitive**	***n* (%)**
absent	13 (25)
mild	9 (18)
moderate	11 (22)
severe	18 (36)
**FSMC motor**	***n* (%)**
absent	8 (16)
mild	8 (16)
moderate	6 (12)
severe	29 (57)
**FSMC total**	***n* (%)**
absent	10 (20)
mild	6 (11)
moderate	9 (18)
severe	26 (51)

**Table 4:** Sociodemographic and clinical characteristics and fatigue scores.

	**Motor fatigue**		**Cognitive fatigue**		**Total fatigue**	
	*r*		*r*		*r*	
Age	0.13		−0.01		0.06	
Time since diagnosis	0.14		0.07		0.11	
	**Motor fatigue**		**Cognitive fatigue**		**Total fatigue**	
	mean ± SD	*t*	mean ± SD	*t*	mean ± SD	*t*
Gender						
Women	34.5 ± 8.6	3.4 *	32.1 ± 9	4.1 **	66.5 ± 16.5	4.1 **
Men	24.8 ± 8.7		22.1 ± 6.7		46.8 ± 14	

* *p* < 0.01; ** *p* < 0.001.

**Table 5:** Correlational analysis between fatigue subscales and psychological characteristics of the sample and partial correlation with anxiety/depression symptoms.

	FSMC Motor Fatigue		FSMC Cognitive Fatigue		FSMC Total Fatigue	
	Spearman Correlation	Partial Correlation	Spearman Correlation	Partial Correlation	Spearman Correlation	Partial Correlation
**Illness perception** (B-IPQ)						
Consequences (B-IPQ 1)	0.72	0.67	0.56	0.48	0.67	0.61
Timeline (B-IPQ 2)	0.18	0.11	0.16	0.03	0.19	0.09
Personal control (B-IPQ 3)	−0.11	−0.17	−0.16	−0.18	−0.13	−0.17
Treatment control (B-IPQ 4)	−0.16	−0.20	0.01	0.05	−0.07	−0.06
Identity (B-IPQ 5)	0.72	0.64	0.53	0.40	0.66	0.55
Concern (B-IPQ 6)	0.42	0.30	0.33	0.19	0.41	0.28
Coherence (B-IPQ 7)	0.14	0.19	0.15	0.22	0.15	0.23
Emotional response (B-IPQ 8)	0.24	0.14	0.28	0.17	0.29	0.18
B-IPQ TOT	0.58	0.50	0.45	0.31	0.54	0.43
**Resilience** (CD-RISC)	−0.41	−0.25	−0.25	−0.06	−0.35	−0.17
**Mindfulness** (FFMQ)						
Observe	0.16	0.26	0.19	0.29	0.17	0.29
Describe	−0.18	−0.00	−0.37	−0.23	−0.31	−0.15
Awareness	−0.32	−0.14	−0.43	−0.25	−0.39	−0.20
Non-judge	−0.39	−0.24	−0.49	−0.36	−0.48	−0.34
Non-react	−0.54	−0.44	−0.45	−0.25	−0.54	−0.38
Total score FFMQ	−0.50	−0.27	−0.59	−0.39	−0.59	−0.37

**Table 6:** Seemingly unrelated regression.

Equation	Obs	Params	RMSE	*R*-Squared	*chi* ^2^	*p* > *chi*^2^
**FSMC_motor**	51	3	6.589737	0.5129	54.71	0.0000
**FSMC_cognitive**	51	3	6.485902	0.5195	54.57	0.0000
	**Observed coefficient**	**Bootstrap Std. Err**	***z***	***p* > |*z*|**	**Normal-Based [95% Conf. Interval]**	
**FSMC_motor**						
female	6.68368	2.19414	3.05	0.002	2.383245	10.98412
HADS_Depression	0.9040935	0.2786286	3.24	0.001	0.3579915	1.450196
BIPQ	2.008766	0.7372678	2.72	0.006	0.5637481	3.453785
_cons	11.11493	3.698706	3.01	0.003	3.865597	18.36426
**FSMC_cognitive**						
female	7.633537	2.087748	3.66	0.000	3.541627	11.72545
HADS_Anxiety	777889	0.2953892	2.63	0.008	0.1989368	1.356841
FFMQ_tot	−0.180669	0.1013753	−1.78	0.075	−0.3793609	0.018023
_cons	31.19096	9.378832	3.33	0.001	12.80879	49.57313

## Data Availability

The data and materials used during the current study are available from the corresponding author (V.D.) on reasonable request.

## Appendix A

### Appendix A.1. Descriptive Statistics of FSMC Scales, by Gender

**Table A1:** Normality assessment via skewness and kurtosis.

	Male			Female		
	Skewness	Kurtosis	S-K Test (*p*-Value)	Skewness	Kurtosis	S-K Test (*p*-Value)
FSMC-cognitive fatigue	−0.34	1.93	1.23 (0.54)	−0.03	2.09	2.67 (0.26)
FSMC-motor fatigue	0.05	1.80	1.40 (0.50)	−0.26	1.91	5.61 (0.06)
FSMC-total fatigue	−0.27	1.88	1.25 (0.54)	−0.19	2.17	2.19 (0.33)

**Table A2:** Homoscedasticity analysis, by using the variance ratio test.

	Male	Female		
	Mean (SD)	Mean (SD)	*F* Test (dof)	*p*-Value
FSMC-cognitive fatigue	22.08 (6.7)	32.06 (9.0)	0.56 (11.38)	0.30
FSMC-total fatigue	46.83 (14.0)	66.58 (16.5)	0.72 (11.38)	0.58

### Appendix A.2. Collinearity Diagnostics

**Table A3:** Collinearity diagnostics for regression model predictors.

Variable	VIF	√VIF	Tolerance	*R* ^2^
Gender (female)	1.16	1.08	0.8648	0.1352
HADS_D	1.60	1.27	0.6241	0.3759
HADS_A	1.84	1.36	0.5423	0.4577
BIPQ	1.43	1.20	0.6970	0.3030
FFMQ	1.64	1.28	0.6116	0.3884
Mean VIF	1.53			
